# Supplementary material for: Merits of hiPSC-Derived Cardiomyocytes for In Vitro Research and Testing Drug Toxicity
Source: Biomedicines. 2022 Oct 31;10(11):2764. doi: 10.3390/biomedicines10112764 (PMC9687838; doi:10.3390/biomedicines10112764)
Supplement: Supplementary file 1 [file biomedicines-10-02764-s001.zip › biomedicines-1965623-supplementary.pdf]

Supplementary data

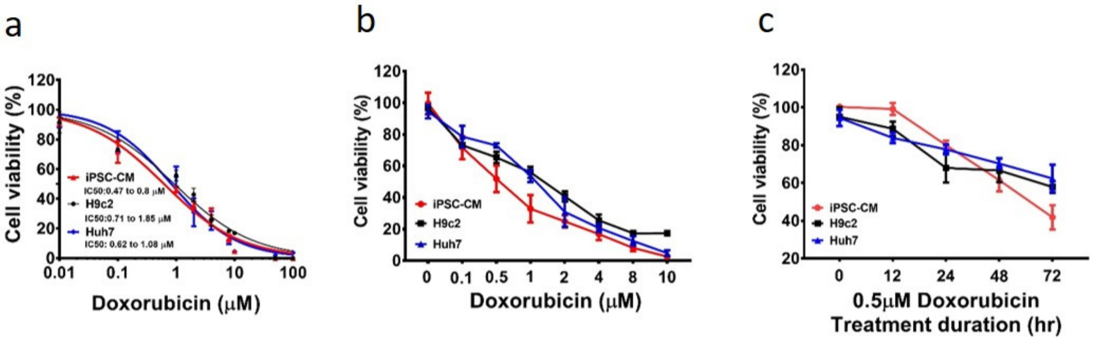

Figure S1. Doxorubicin-induced toxicity in hiPSC-differentiated cardiomyocytes (iPSC-CMs), and H9c2 and Huh7 cells. (**a**) IC50 of doxorubicin treatment in iPSC-CMs, H9c2, and Huh7 cell after 72 h. (**b**) Effect of doxorubicin dose on cell viability after treatment for 72 h. (**c**) Effect of doxorubicin treatment duration on cell viability. Each data point represents the mean ± SEM (n = 3).

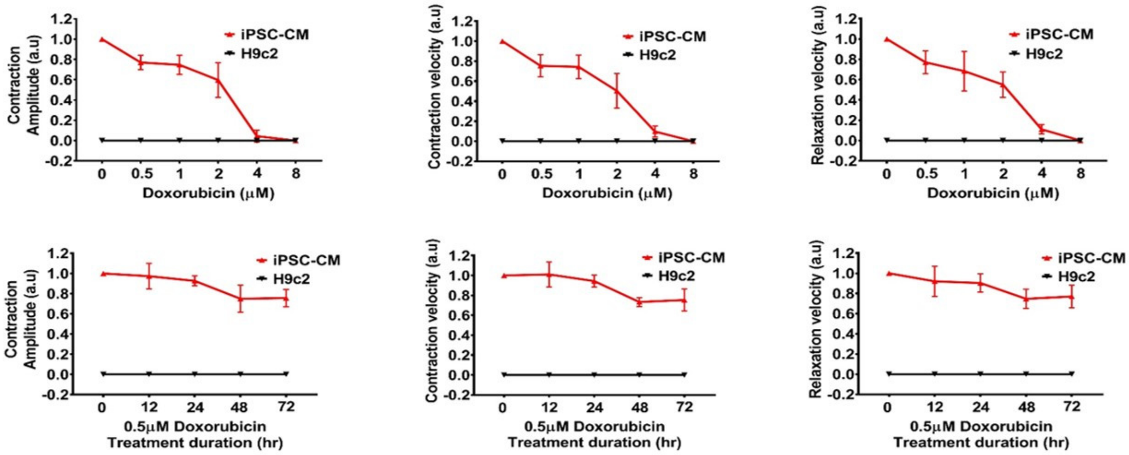

Figure S2. Contractile dysfunction in doxorubicin-treated hiPSC-differentiated cardiomyocytes (iPSC-CMs). (**a-c**) Effect of doxorubicin dose (0, 2, 4, 8, and 12 μM) on muscle motion of iPSC-CMs and H9c2 cells after treatment for 24 h. (**a**) Contraction amplitude, (**b**) contraction velocity, and (**c**) relaxation velocity. (**d-f**) Effect of time on muscle motion of iPSC-CMs and H9c2 cells treated with 0.5 μM of doxorubicin for 72 h. (**d**) Contraction amplitude, (**e**) contraction velocity, and (**f**) relaxation velocity. Each data point represents the mean ± SEM (n = 3).

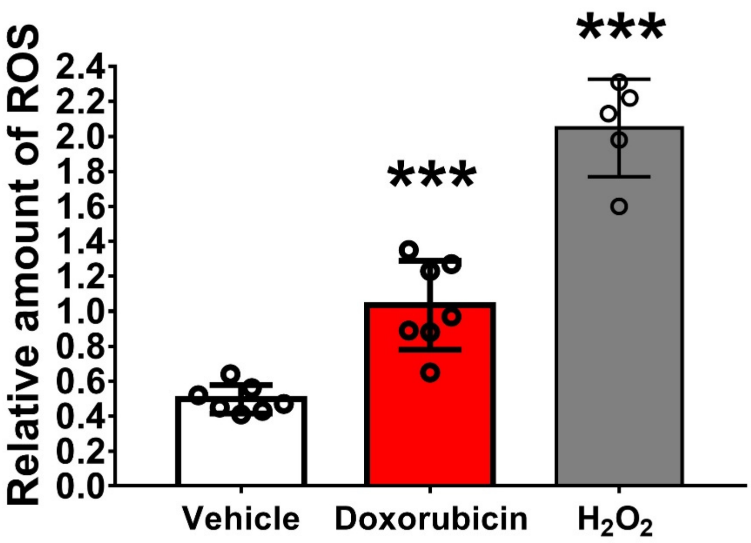

Figure S3. Quantification of reactive oxygen species (ROS) generated in doxorubicin-treated human induced pluripotent stem cell-derived cardiomyocytes. *** Significantly different from vehicle ($p < 0.001$).
